# Chemical Profile and Biological Activity of Cherimoya (*Annona cherimola* Mill.) and Atemoya (*Annona atemoya*) Leaves

**DOI:** 10.3390/molecules25112612

**Published:** 2020-06-04

**Authors:** Giuseppe Mannino, Carla Gentile, Alessandra Porcu, Chiara Agliassa, Fabio Caradonna, Cinzia Margherita Bertea

**Affiliations:** 1 Department of Life Sciences and Systems Biology, Innovation Centre, Plant Physiology Unit, University of Turin, Via Quarello 15/A, 10135 Turin, Italy; giuseppe.mannino@unito.it (G.M.); alessandra.porcu90@gmail.com (A.P.); cinzia.bertea@unito.it (C.M.B.); 2 Department of Biological, Chemical and Pharmaceutical Sciences and Technologies (STEBICEF), University of Palermo, Viale delle Scienze, 90128 Palermo, Italy; fabio.caradonna@unipa.it; 3 Department of Agricultural, Forest, and Food Sciences, University of Turin (DISAFA), Largo Paolo Braccini 2, 10095 Grugliasco, Italy; chiara.agliassa@unito.it

**Keywords:** antioxidants, HPLC-DAD-MS/MS, polyphenols, alkaloids, antiproliferative activity

## Abstract

*Annona cherimola* (Cherimoya) and *Annona atemoya* (Atemoya) are tropical plants known for their edible fruit. Scientific data suggest that their leaves, used in traditional medicine in the form of teas or infusions without evidence of toxicity, contain several bioactive compounds. However, only *Annona muricata* among all the *Annona* species is currently used in the nutraceutical field, and its dried leaves are marketed for tea preparation. In this work, we explored the nutraceutical potential of Atemoya and Cherimoya leaves, by evaluating their chemical profile and functional properties. Phytochemical analyses showed large amounts of phenolic compounds, in particular proanthocyanidins, and identified 18 compounds, either flavonoids or alkaloids. Concerning biological activity, we found antioxidative properties correlated with polyphenols, and antiproliferative activity against HeLa and HepG2 cell lines correlated with alkaloids. The obtained results demonstrate the potential use of *Annona cherimola* leaves for the preparation of dietary supplements aimed to promote the physiological redox balance. Moreover, the varietal comparison suggests that two commercial cultivars (*Campas* and *White*) and the local *Torre 1*, better suit this purpose. On the other hand, among the studied cultivars, *Campas* and *Torre 1* are also the richest in alkaloids and, in consideration of the anti-proliferative properties of their extracts, dietary supplements based on these cultivars might also have chemo-preventive effects.

## 1. Introduction

Plants are the most important sources of biologically active compounds [1]. Since ancient times, numerous plant preparations have been used in folk medicine for the treatment of several diseases [2] and presently, nutraceutical, pharmaceutical, and cosmetic industries are increasingly interested either in pure phytochemicals or plant preparations. Consistently, the plant preparation market, in terms of value, is projected to reach around USD 86.74 Billion by 2022, with the pharmaceutical sector as the largest market share, followed by the nutraceutical field for nutritional supplement preparations. Interestingly, plant preparation for medicine, food and beverages, and cosmetics are largely based on plant leaves. Indeed, although bioactive compounds are stored as secondary metabolites in the whole plant, leaves are the largest accumulators of such compounds among all plant organs. Furthermore, being readily available in quantities suitable for industrial purposes, leaves are an ideal source of nutra-pharmaceutical compounds, especially considering their low nutritional value and, at the same time, their nature of agricultural waste.

Approximately half the flowering plant species lives in the tropical forests [3], and in recent years, the interest in these species greatly increased, especially in consideration of the nutritional and nutraceutical value of some tropical fruit [4,5,6]. On the other hand, many studies have evaluated the phytochemical profile of different parts of several tropical plants, and an increasing amount of scientific evidence suggested their potential use for applications in the nutraceutical, cosmetic and pharmacological field [7].

The *Annona* genus is part of the *Annonaceae* family, which comprises more than 119 species, and among them, 109 are native of tropical America [8]. *Annona cherimola* Mill (Cherimoya), which literally means “cold seeds”, is a small domesticated tree that produces heart-shaped and conical edible fruit. The ovate-lanceolate leaves are glabrous on the ventral surface and pubescent dorsally [8]. The commercial value of Cherimoya is mainly related to its fruit, essentially destined for fresh consuming. However, Cherimoya fruit is traditionally used for the treatment of several diseases [9]. Recently, it has been shown that different parts of Cherimoya possess an interesting phytochemical profile, with high content in polyphenols and alkaloids [10]. In particular, similarly to other species belonging to *Annona* genus [11,12,13,14,15], leaves of Cherimoya were found to be a potential source of bioactive compounds [16] and are currently ingredients in traditional medicine preparation and folk teas for the treatment of gastric, intestinal, cardiovascular, skin, and eye diseases [17].

Despite the long usage history of these preparations without evidence of toxicity and the proven presence of bioactive compounds, the nutraceutical potential of Cherimoya leaves has been poorly explored. Today, these leaves are just discarded as agricultural waste, usually in considerable quantities. Among all the *Annona* species, only *Annona muricata* is currently used in the nutraceutical field. Since its high content of antioxidants with potential protective effects on health, graviola tea, obtained from the dried leaves of *Annona muricata*, is indeed on the market as a food supplement. 

Despite the attractive organoleptic qualities of their fruit, Cherimoya is an under-used species in the native country of Latin America. Today, with a total cultivated area of around 3.000 ha, Spain is the world’s largest producer [18]. Due to the increase of marketable interest for its fruit, the cultivation of this plant was extended to different areas, including other Mediterranean regions, such as Sicily [19]. Moreover, in order to develop tastier fruit and a better adaptation to specific climatic conditions, new cultivars were introduced and the hybrid of *A. cherimola* and *A. squamosa* started to be produced and marketed with the name of Atemoya [20].

In this study, we explored the nutraceutical potential of leaves of several *Annona* species. In particular, we investigated the chemical composition of leaves of seven *A. cherimola* cultivars (*Campas*, *Chaffey*, *Daniela*, *Fino de Jete*, *Torre1*, *Torre2*, and *White*), and of the commercial hybrid *A. atemoya* grown in Sicily. In addition, in order to prospect protective effects derived from the consumption of teas, infusions or drinks obtained from these leaves, antioxidative and antiproliferative activities were also evaluated.

## 2. Materials and Methods

### 2.1. Standards and Chemicals

The solvents used for extraction process and chemical analysis were of analytical grade and were purchased from VWR International (Radnor, PA, USA). 2,2′-azinobis(3-ethylbenzothiazoline-6-sulfonic acid) diammonium salt (ABTS), 2,2-diphenyl-1-picrylhydrazyl (DPPH·), 4-(Dimethylamino)cinnamaldehyde (DMAC), Folin-Ciocalteau’s reagent, 6-hydroxy-2,5,7,8-tetramethylchroman-2-carboxylic acid (Trolox), Sodium Acetate, 2,4,6-Tri(2-pyridyl)-s-triazine (TPTZ), FeCl_3_, potassium persulfate, Nuciferine, phenolic standards (Catechin, Quercetin 3-*O*-rutinoside-7-*O*-glucoside, Epicatechin, Quercetin 3-*O*-rutinoside, Kaempferol-3-Galactoside-7-Rhamnoside, Quercetin-3-*O*-glucoside, Kaempferol-3-*O*-glucoside, Apigenin 8-*C*-glucoside, Luteolin-3-Galactoside-7-Rhamnoside) were purchased from Sigma Aldrich (Gillingham, UK). RPMI, fetal bovine serum (FBS), phosphate buffered saline (PBS), l-glutamine solution (200 mM), trypsin-Ethylenediaminetetraacetic acid (EDTA) solution (170,000 U/L trypsin and 0.2 g/L EDTA) and penicillin-streptomycin solution (10,000 U/mL penicillin and 10 mg/mL streptomycin) were purchased from Lonza (Verviers, Belgium).

### 2.2. Plant Material and Preparation of Leaf Extract

The trees of both *Annona atemoya* and of the seven different cultivars of *Annona cherimola* (var. *Campas*, *Chaffey*, *Daniela*, *Fino de Jete*, *Torre1*, *Torre2*, and *White*) were grown in Vivai Torre (Milazzo, Sicily, Italy; 38°19’ N, 15°24’ E; 20 m a.s.l.). After the collection, leaves were dried in oven at 50 °C for 16 h, and then crushed. The obtained powders were extracted with a 75:25 (*v*/*v*) ethanol:water solution, using 1:20 (*w*/*v*) extraction ratio, for 3 days. The samples were kept in the dark at room temperature throughout the entire process. After centrifugation (10 min at 10,000× *g*, at 4 °C) and filtration through a Millex HV 0.45 μm filter (Millipore, Billerica, MA, USA), the supernatants were recovered and stored at −80 °C until analysis. For each sample, the extraction was performed in triplicate.

### 2.3. Total Phenolic Content

The total phenolic content (TPC) was determined by the Folin-Ciocalteu’s method [21]. An external calibration curve of gallic acid (GA) was used to quantify these compounds in the different samples. The results were expressed as mg of gallic acid equivalent (GA equivalent) per 100 g of d.wt weight. All measurements were performed in three different replicates.

### 2.4. Total Proanthocyanidin Content

The total proanthocyanidin content (tPAC) was evaluated by DMAC assay, as previously described by Prior and colleagues [22] with minor modifications [23]. The tPAC was quantified using an external calibration curve of pure PAC-A2 type as standard (Extrasynthese^®^, France). The results were expressed as mg PAC-A2 type per 100 g of d.wt weight. All measurements were performed in three different replicates.

### 2.5. Identification and Quantification of Phenolic and Alkaloid Compounds by HPLC-ESI-DAD-MS/MS

The chemical portioning of the leaf ethanolic extracts was performed by High Performance Liquid Chromatography (HPLC) coupled both with a Diode Array Detector (DAD) and Mass spectrometer (MS). The analysis was conducted using Dionex UltiMate^®^3000 Rapid Separation LC system (Thermo Fischer Scientific, San Jose, CA, USA), with an auto-sampler controlled by Chromeleon 7.2 Software (Thermo Fisher, Bremen, DE and Dionex Softron GmbH, Germering, DE). The column was a Phenomenex Luna C18 (3.00 μm, 150 mm × 3.0 mm i.d., Phenomenex, Torrance, CA, USA), packed with core-shell particles of 2.5 μm. The flow rate was set at 200 μL·min^−1^ at 25 °C.

#### 2.5.1. Analysis of Alkaloids

**Figure 1:**
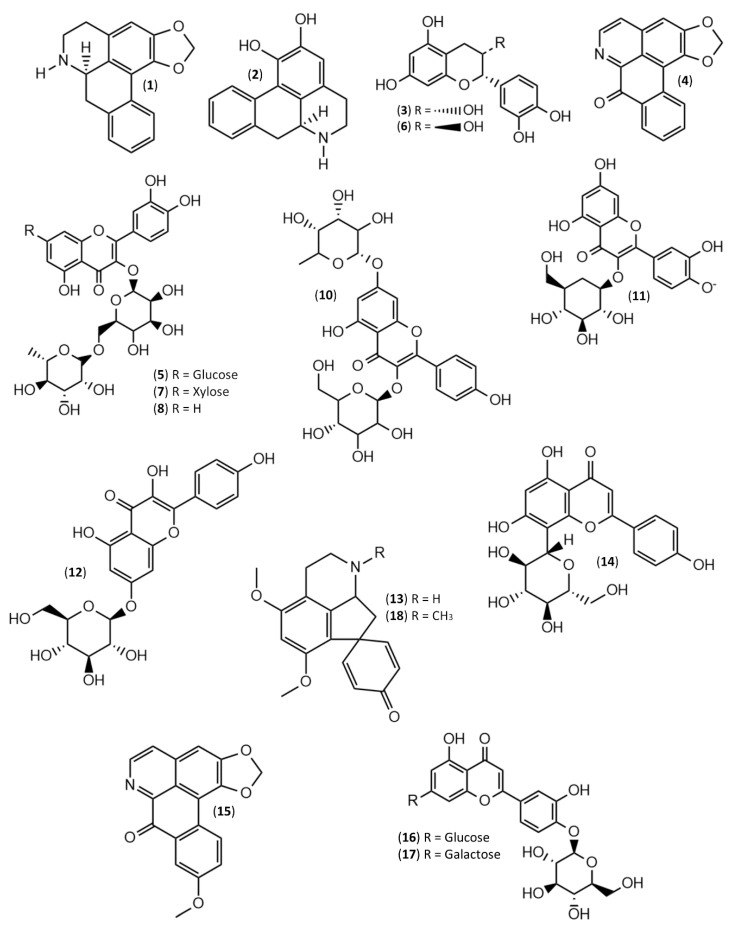
Chemical Structures of the phenolic and alkaloid compounds characterized and quantified in the seven extracts of Cherimoya and Atemoya leaves.

The analysis of alkaloid compounds was performed using the chromatographic conditions previously described by Vigliante and colleagues for the identification and quantification of *N*-Containing Compounds in ethanolic extracts of *Griffonia simplicifolia* [24]. Briefly, the binary solvent system was MilliQ H_2_O acidified with 0.1% *v*/*v* formic acid (Solvent A) and acetonitrile acidified with 0.1% *v*/*v* formic acid (Solvent B). The samples were separated by an isocratic gradient of 97% A and 3% B. To clean the column from other interferences before the next injection, the concentration of B was slowly raised and maintained at 3% for 5 min. A typical chromatographic profile of the samples is shown is Supplementary Figure S1 (Panel A). Alkaloid identification was performed considering the retention time and the fragmentation pattern of each compound and by comparing them with those previously reported in the literature. Finally, due to the unavailability in commerce of pure standard, a tentative-quantification was performed using an external calibration curve of nuciferine at opportune concentrations (0.01–1 μg/mL). The chemical structures of the identified alkaloid compounds was reported in Figure 1.

#### 2.5.2. Analysis of Polyphenolic Compounds

The analysis of polyphenolic compounds was performed using the chromatographic condition previously described by Gentile and colleagues for the identification and quantification of phenolic compounds in ethanolic extracts of *Mangifera indica* [4]. Briefly, the binary solvent system was MilliQ H_2_O acidified with 0.1% *v/v* formic acid (Solvent A) and acetonitrile acidified with 0.1% *v/v* formic acid (Solvent B). The elution was performed according to the following method: 0–10 min 5% B; 10–13 min linear increase to 95% B; 13–20 min hold 95% B; 21 min linear decrease 5% B and 22 min coming back to the initial conditions. A typical chromatographic profile of the samples is shown is Supplementary Figure S1 (Panel B). Polyphenol identification was performed by analyzing each compound retention time and fragmentation pattern, and by comparing them either to those already collected in the literature or to those of pure compounds. Moreover, quantification was performed using opportune concentrations of each pure compound (obtained by Sigma Aldrich, St. Louis, MO, USA) (0.01–1 μg/mL) in an external calibration curve. Finally, due to the commercial unavailability of pure standards for **7** and **17**, their quantification was performed using the calibration curves of **5** and **16**, respectively. The chemical structures of the identified phenolic compounds was reported in Figure 1.

### 2.6. Antioxidant Activity

Antioxidant activity of the ethanolic extracts from leaves of the plants used in this study was measured by ABTS [25], DPPH [26], and ferric reducing antioxidant power (FRAP) [27] assays with minor changes. 

#### 2.6.1. ABTS

7 mM of 2,2’-azino-bis(3-ethylbenzothiazoline-6-sulphonic acid (ABTS) was dissolved in water and left to react with 2.45 mM potassium persulfate. After 16 h of incubation in the dark, the ABTS^+^ solution was diluted with ethanol until an absorbance of 0.70 at 734 nm at room temperature was obtained. Samples and 6-hydroxy-2,5,7,8-tetramethylchroman-2-carboxylic acid (Trolox), used as reference standard, were incubated at different and appropriate dilutions. After 5 min of incubation, the decolorization of the mixture was read at 734 nm.

#### 2.6.2. DPPH

0.1 mM of the radical 2,2-diphenyl-1-picrylhydrazyl (DPPH^·^) solution was prepared in ethanol and 1 mL of this solution was added to different concentrations of the sample. The mixture was shaken vigorously and left to stand for 30 min in the dark, and the absorbance was measured at 517 nm. Samples and Trolox, used as reference standard, were incubated at different appropriate dilutions. After 20 min of incubation time at room temperature the decolorization of the mixture was read at 517 nm.

For both ABTS and DPPH assays, antioxidant activity was calculated using the following equation:

(1) $$\mathrm{AA}\%=\frac{A_{\mathrm{blank}}-A_{\mathrm{sample}}}{A_{\mathrm{blank}}}\;\times \;100$$

where: AA% is the percentage of color reduction of the reagent; A_blank_ is the absorbance of blank and A_sample_ the absorbance of the sample at 517 nm. Trolox was separately assayed following the same procedure employed for the sample, and the results were expressed as mmol of Trolox equivalent (TE) per 100 g of d.wt weight. All measurements were performed in three different replicates.

#### 2.6.3. FRAP

0.3 M acetate buffer (pH 3.6), 10 mM 2,4,6-Tri(2-pyridyl)-s-triazine (TPTZ) and 20 mM FeCl_3_ were mixed together in 8:1:1 (*v*/*v*/*v*) ratio. The mix solution was incubated at 37 °C for 10 min with an appropriate dilution of samples. The absorbance was then read at 595 nm. An external calibration curve of Trolox was used for the quantification, and the results were expressed as mmol TE per 100 of d.wt weight. All measurements were repeated three times.

### 2.7. Antiproliferative Activity

#### 2.7.1. Cell Cultures

HeLa (human cervical cancer) and HepG2 (human hepatocarcinoma) cells were obtained from American Type Culture Collection (Rockville, MD, USA). Cells were cultured in RPMI supplemented with 5% FBS, 2 mM l-glutamine, 50 IU/mL penicillin, and 50 µg/mL streptomycin. The cells were trypsinized approximately between the 75–85% of their confluence, and exponentially growing cells were used for the evaluation of antiproliferative activity.

#### 2.7.2. MTT Assay

The 3-[4,5-dimethylthiazole-2-yl]-2,5-diphenyltetrazolium bromide (MTT) was performed as previously described [28,29]. Briefly, after the seeding of the cells into 96-well plates, they were incubated for 24 h. Different solutions of the hydrophilic extracts were added to the cells, and then incubated again for 48 h. After the incubation time, a medium containing 0.5 mg/mL of MTT reagent was added to each well, and then discarded after a 3 h incubation at 37 °C. The formazan salt formed in the cells was dissolved in dimethyl sulfoxide (DMSO). The absorbance at 570 nm of MTT-formazan was measured in a microplate reader (GloMax^®^ Multidetection Plate Reader, Promega^®^) and the percentage of growth (PG) with respect to untreated cells was calculated in accordance with the following equation:

(2) $$\mathrm{PG}\%=\frac{OD_{\mathrm{test}}-OD_{\mathrm{tzero}}}{OD_{\mathrm{ctr}}-OD_{\mathrm{tzero}}}\;\times \;100$$

where OD_test_ is the average of optical density measurements after the exposure of cells to the tested extract; OD_tzero_ is the average of optical density measurements before the desired period of time; OD_ctr_ is the average of optical density measurements after the desired period of time with no exposure of cells to treatment. Each extract concentration necessary to induce 50% growth inhibition (GI_50_) was calculated from concentration-response curves using linear regression analysis by fitting the tested concentrations that give PG values above and below the reference value. Each result is the mean value of five separate experiments.

Finally, in order to exclude potential cytotoxic effects of the extracts at the concentration range used for our experiments, Trypan blue exclusion method was employed.

### 2.8. Statistical Analysis

All statistical analyses were performed using SPSS package ver. 25. Spectrophotometric, analytical, and biological data were tested for differences among the different species using the one-way analysis of variance (ANOVA; general linear model) followed by Tukey’s multiple range test for *p* ≤ 0.05. Alkaloid and polyphenolic profile obtained by HPLC-DAD-MS/MS were analyzed also via principal component analysis (PCA).

## 3. Results and Discussion

### 3.1. Identification and Quantification of Polyphenolic Compounds in Extracts of Cherimoya and Atemoya Leaves

Polyphenols are the most widely distributed phytochemicals and contribute significantly to the biological activity of plant extracts [28]. Total phenolic compounds in leaf extracts of the observed cultivars of Cherimoya and Atemoya were quantified by Folin-Ciocalteu method (Table 1).

Significant differences were found among the samples. The values varied in a wide range with an average value of 599.55 ± 132.37 mg GA equivalent per 100 g of d.wt material. Our results are similar to previous TPC values evaluated on leaf extracts of *A. cherimola* [16], *A. muricata* [15], and *A. squamosa* [14]. In our experimental conditions, the highest value was found in *Torre2*, followed by *White* and *Fino de Jete*, whereas *Torre1* showed the lowest content.

Among polyphenols, PACs are found in fruit, leaves and roots of several plants, and they are the most abundant flavonoids in the human diet [30,31]. Several epidemiological studies connect PACs consumption to health benefits. In particular, they were reported to have antioxidant, anti-inflammatory, antiallergic, and anticancer properties and positive effects on cardiovascular function [30,32]. In this work, we quantified the tPAC in the leaves of the observed samples of Cherimoya and Atemoya through DMAC assay (Table 1). The obtained data displayed a great variability among both the studied genotypes and the cultivars, and in particular the samples *Torre2* and *Campas* scored the highest concentrations of PACs, while the lowest value belonged to *Torre1*. The Pearson Correlation Coefficient (PCC) showed a high correlation between the tPAC and TPC (ρ = 0.81), suggesting that PACs contributed significantly to TPC of the observed leaf extracts.

To identify and quantify single phenolic compounds in our samples, HPLC-DAD-ESI-MS/MS was employed. We identified 11 flavonoid compounds in the analyzed extracts (Figure 1). Among the identified flavonoids, six were flavonols [Quercetin-3-*O*-rutinoside-7-*O*-glucoside (**5**), Quercetin-3-*O*-rutinoside-7-*O*-pentoside (**7**), Quercetin-3-*O*-rutinoside (**8**), Kaempferol-3-Galactoside-7-Rhamnoside (**10**), Quercetin-3-*O*-glucoside (**11**) and Kaempferol-3-*O*-glucoside (**12**)], three were flavones (Luteolin-3-Galactoside-7-Rhamnoside (**14**), Luteolin-3-Glucoside-7-Rhamnoside (**16**) and Apigenin-8-*C*-glucoside (**17**)] and two were flavan-3-ols [Catechin (**3**) and Epicatechin (**6**)). Our results are in agreement with Dìaz-De-Cerio and colleagues that reported for the first time the polyphenolic profile of the leaves of *A. cherimola* var. *Fino de Jete* [16].

Although we found a comparable qualitative profile among the different genotypes and cultivars, our data showed significant (*p* ≤ 0.05) differences in the content of the individual identified compounds (Table 2). In all samples, the most abundant compounds were the two flavonols **8** and **11**, which represented a percentage between 80% and 93% of the total weight of the identified polyphenols. Concerning the other identified polyphenols, the amount exceeded the 5% of the total weight of the identified polyphenols only in a few samples. In particular, the flavonols **10** found in *A. atemoya* and *Daniela*, **12** in *Daniela*, and the flavone **16** in *Torre 1*. Moreover, **5** was not detected in *Cheffey*, *Daniela* and *Torre1.* The latter cultivar, *Torre1*, contained neither **3** nor **6**. In addition, we conducted a target analysis for the identification and quantification of PACs via HPLC-DAD-MS/MS, but no signal was recorded. Considering the limit of our HPLC-MS/MS system in the detection of compounds with high molecular weight (MW), the contrast between BL-DMAC and HPLC-MS/MS data suggested the presence of PACs with high polymerization grade.

### 3.2. Identification and Quantification of Alkaloid Compounds in Extracts of Cherimoya and Atemoya Leaves

Phytochemical characterization of the leaf extracts showed six alkaloid compounds (Figure 1): two aporphines (Anonaine (**1**) and Asimilobine (**2**)), two oxoaporphines (Liriodenine (**4**) and Lanuginoside (**15**)) and two proaporphines ((Stepharine (**13**) and Pronuciferine (**18**)). Although a similar alkaloid profile was observed in the leaves of others species belonging to the *Annona* genus [33,34,35], including *A. atemoya* [36], to the best of the author knowledge, to date data regarding their quantification have not been collected. In general, small qualitative differences were found among the cultivars under study (Table 2). In particular, while **13**, **15** and **18** were always displayed in all the cultivars, the aporphine **1** was only present in *Campas*, *Fino de Jete* and *Torre2*. On the contrary, the aporphine **2** was absent in *Chaffey*, *Torre1*, and *Torre2*. Finally, no trace of oxoaporphine **4** was found in *Daniela* and *Torre2*.

In all cultivars, **15** was the most concentrated, followed by **13** and **18**. In particular, the two identified oxoaporhines (**4** and **15**) constituted more than the 68% of the total dried weight of the identified alkaloids among Cherimoya cultivars. On the contrary, the two aporphines (**1** and **2**) were found in the lowest concentration, representing only about the 1% of the total weight of the identified alkaloids. Alkaloid profile of Atemoya leaves was significantly different from that observed in Cherimoya cultivars. In particular, in the commercial hybrid the amount of oxoaporphine (**4** and **15**) and proaporphine (**13** and **18**) was perfectly comparable.

### 3.3. Principal Component Analysis (PCA) Discriminates the Different Genotypes

**Figure 2:**
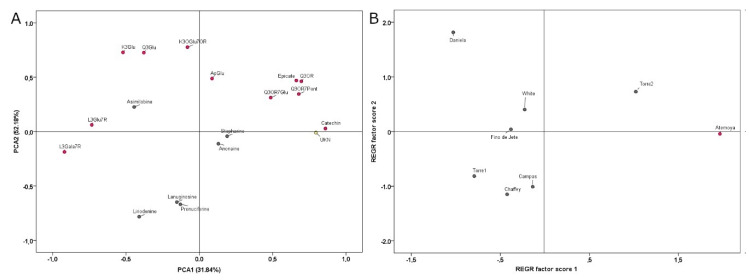
Scatter plot of the principal components (PC1 and PC2) factor scores calculated on the PCA of phenolic and alkaloid compounds of the seven extracts of Cherimoya and Atemoya leaves. PCA was performed using the data matrix in Table 2 obtained with HPLC-DAD-ESI-MS/MS analysis. Panel A represent the partitioning of the different compounds based on PC1 and PC2 factor scores. Panel B represent the partitioning of the different genotypes and cultivars according to their chemical composition.

Data resulted from the quantification of both polyphenolic and alkaloid compounds obtained from HPLC-DAD-ESI-MS/MS (Table 2) were employed for the Principal Component Analysis (PCA) (Figure 2). PCA explained 52.18% and 31.84% of the total variance for PC1 and PC2, respectively. Positive factor scores discriminated *Torre2* from others for its high content of **3**, **7**, **8**, **13** and **14**. On the other hand, *Campas*, *Chaffey* and *Torre1* were discriminated for negative factor scores due to the concentration of **4**, **15**, **16** and **18**. Negative PC1 and positive PC2 factor scores grouped *Daniela*, *White* and *Fino de Jete* for the presence of the aporphine **2** and their characteristic content of **11**, **12** and **17**. Finally, the commercial hybrid *Annona atemoya* was completely separated from the *Annona cherimola* cultivars due both to the presence of the non-identified compound **9** (MW 478, MS/MS 323) and the absence of **1** and **2**.

### 3.4. Evaluation of Antioxidant Properties in Extracts of Cherimoya and Atemoya Leaves

Considering the implication of oxidative stress phenomena in several diseases, many plant materials are largely investigated for their antioxidant properties [4,5,28]. Currently, in order to evaluate the antioxidant properties of plant extracts and their mechanism of action, several in vitro methods are employed [37].

Biological activity, including the antioxidant activity of fruit belonging to *Annona* genus was investigated in several scientific studies [38,39,40], but reducing potential of these plant leaf extracts was poorly characterized [41]. Here, we evaluated for the first time the antioxidant activity, including the radical scavenging (ABTS and DPPH) and the metal reducing (FRAP) activities of leaves of *A. cherimola* and *A. atemoya*. The obtained data are reported in Table 3. Our results showed a significant (*p* ≤ 0.05) variability in antioxidant capacities of the studied extracts, but also strong differences based on the assay used for the estimation. As a general trend, ABTS showed always the lowest values, and in particular, DPPH and FRAP values were 23– fold higher than ABTS. The observed differences between the assays could be explained by the dissimilarity in pH, in hydrophilicity of the reaction mixtures, and in the reducing activity of antioxidant compounds present in the extracts [37]. However, independently from the assay employed for the estimation, *Torre2* displayed the best activity, whereas *Torre1* the lowest one. Moreover, we recorded a strong antioxidant activity also for Atemoya, *Campas*, and *White*.

When we compared the antioxidant properties with polyphenolic content, we found a high correlation between antioxidant activity and TPC (ρ_ABTS_ = 0.94; ρ_DPPH_ = 0.89; ρ_FRAP_ = 0.97), suggesting that polyphenols strongly contribute to antioxidant properties of the extracts. Moreover, we found also a very high correlation between FRAP and tPAC (ρ_FRAP_ = 0.96). Considering that ferric reducing ability of polyphenols can involve also early metal complexation through bidentate binding sites, this correlation may be due to the presence of compounds with free meta-oriented hydroxyl groups [4,5]. On the other hand, no correlation was found between antioxidant activity and alkaloid content.

### 3.5. Evaluation of Antiproliferative Activity in Extracts of Cherimoya and Atemoya Leaves

The consumption of plant material has also been associated with the prevention of several chronic diseases, and a constant supply of phytochemical-containing plants is essential to fortify defensive mechanism and bring health benefits [42]. In addition, plant extracts or individual purified polyphenol or alkaloid compounds display bioactivity, including antiproliferative activity, in several in vitro models [28,39]. In particular, alkaloids and their derivatives, are today the most successful anticancer drugs on the market [43].

**Figure 3:**
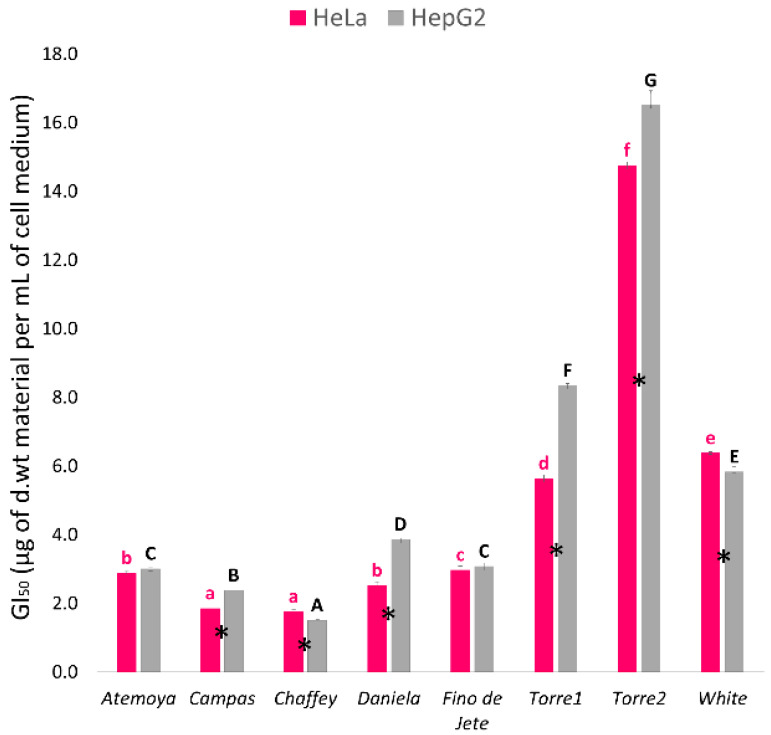
Antiproliferative activity of the seven extracts of Cherimoya and Atemoya leaves against HeLa and HepG2 tumor cell lines. Cell growth was measured by MTT assay after 48 h of treatment with different concentrations of each leaf extract and was expressed as GI50 (µg of d.wt. weight per mL of cell medium). In the histogram, each column represents the mean ± SD of three independent experiments. Among the same series (HeLa or HepG2), different letters indicate significant differences at *p* ≤ 0.05 as measured by Tukey’s multiple range test. Letter “a” denotes the highest antiproliferative activity. “*” indicates statistical differences between the two cancer cell lines. For additional statistical analysis information see Supplementary Table S3.

In this work, we tested for the first time the antiproliferative activity of leaf extracts of *Annona* genotypes against human cervical (HeLa) and liver (HepG2) cancer cell lines by MTT assay. For the experiments, we exposed the cells to extract concentration from 0.1 to 20 µg of d.wt material per mL of cell medium. In this concentration range, the cellular membrane integrity was not affected, as determined preliminarily by Trypan blue exclusion method (data not shown). Data obtained from MTT assay are reported in Figure 3. As a general trend, all the tested extracts showed concentration-dependent antiproliferative activity. However, we found a significant (*p* ≤ 0.05) difference between the two tested cell line and among genotypes. Indeed, the antiproliferative activity toward HepG2 cells was almost always higher than toward HeLa cells. In particular, for *Campas*, *Daniela*, *Torre1* and *Torre2* the highest growth inhibitory effect was found against HeLa cells, while for *Atemoya* and *Fino de Jete* was discovered against HepG2. However, *Campas*, *Chaffey*, *Daniela* and *Fino de Jete* showed very high activity with GI_50_ ranging from 1.76 ± 0.06 to 2.87 ± 0.06 and from1.50 ± 0.02 to 3.86 ± 0.03 µg of d.wt per mL of cell medium against HeLa and HepG2, respectively. On the other hand, *Torre1*, *Torre2* and *White* extracts displayed the lowest antiproliferative activity, with a mean value of 8.92 ± 0.08 and 10.22 ± 0.21 µg of d.wt per mL of cell medium, for HeLa and HepG2, respectively.

There is no significant linear relationship between ABTS, DPPH, FRAP, TPC or tPAC and antiproliferative activity expressed as GI_50_ value. This evidence suggests that antioxidant properties, including reducing activity and antioxidant compound content, cannot explain the observed antiproliferative activity of the studied leaf extracts. It is possible that other specific components in the extracts may play an important role. Moreover, our results are in accordance with those reported by Gavamukulya and colleagues, who demonstrated the antiproliferative activity of ethanolic extracts of *Annona muricata* leaves, proposing an anticancer mechanism that excluded the contribution of antioxidant compounds [12]. Finally, it is interesting to note how although the total alkaloid content was not correlated with GI_50_ value (Supplementary Table S5), the antiproliferative activity of the leaf extracts was strongly correlated with **2** (ρ_HeLa_ = −0.99; ρ_HepG2_ = −0.91), averagely correlated with **4** (ρ_HeLa_ = −0.60; ρ_HepG2_ = −0.42), **18** (ρ_HeLa_ = −0.59; ρ_HepG2_ = −0.58) and to the total content of aporphines (ρ_HeLa_ = −0.60; ρ_HepG2_ = −0.53). These last results are similar to those obtained by Volobuff and colleagues who demonstrated a similar chemo-preventive property for a purified and enriched fraction of these same alkaloids obtained from *Annona cacans* extracts [44].

## 4. Conclusions

The results of this study provide useful information on the chemical and functional properties of the leaves of *A. cherimola* and *A. atemoya.* In particular, we found that leaves of these plants contain significant amounts of polyphenols and alkaloids showing antioxidant properties. The obtained results demonstrate the real potential of *A. cherimoya* leaves for applications in the nutraceutical industry, and in particular for the preparation of dietary supplements with the aim to promote the physiological redox balance, important in the prevention of many chronic diseases. Moreover, the varietal comparison suggests that some *A. cherimoya* cultivars better suit this purpose. In particular, the two cultivars with the greatest commercial impact (*Campas* and *White*) and the local one (*Torre 1*), displayed the best polyphenolic composition. On the other hand, the extracts of *Campas* and *Torre 1* not only were the richest in alkaloid compounds, but also displayed the best antiproliferative activity against HeLa and HepG2 cell lines. For this reason, dietary supplements based on the leaves of this cultivars may also display chemo-preventive effects. Overall, even though further studies are needed, our results suggest a real nutraceutical potential of the leaves of *A. cherimola* and *A. atemoya.* These phytochemical-rich plant sources may find applications not only for the preparation of functional foods, such as teas, decoction, infusions, and drinks, but also for the formulation of dietary supplements to improve oxidative defenses, or prevent chronic diseases.

## Figures and Tables

**Table 1:** Total polyphenol content (TPC) and total proanthocyanidin content (tPAC) of the seven extracts of Cherimoya and Atemoya leaves. TPC was measured by Folin-Ciocalteu method and its related values are expressed as mg of gallic acid equivalent (GA equivalent) per 100 g of dried material (d.wt). tPAC was measured by DMAC assay and its related values are expressed as mg of PAC-A equivalent per 100 g of d.wt. Values are expressed as mean (SD) of three experiments carried out in triplicate.

Genotypes	TPC	tPAC
*Atemoya*	586.64 (12.09) ^bcd^	53.94 (1.23) ^d^
*Campas*	639.34 (9.72) ^bc^	132.26 (3.93) ^a^
*Chaffey*	569.72 (26.93) ^cd^	58.94 (1.15) ^c^
*Daniela*	510.06 (82.12) ^d^	20.76 (0.41) ^f^
*Fino de Jete*	650.75 (17.58) ^bc^	42.26 (1.56) ^e^
*Torre1*	362.76 (22.32) ^e^	8.45 (0.65) ^g^
*Torre2*	824.17 (35.67) ^a^	130.21 (2.23) ^a^
*White*	652.99 (3.45) ^b^	75.98 (2.35) ^b^

For each column, different lowercase letters indicate significant differences at *p* ≤ 0.05 as measured by Tukey’s multiple range test. Letter “a” denotes the highest content. For further statistical analysis information see Supplementary Table S1.

**Table 2:** Phenolic and alkaloid compounds identified and quantified (mg per 100 g of d.wt material) in the seven extracts of Cherimoya and Atemoya leaves by HPLC-DAD-MS/MS. Values are expressed as mean (±SD).

#	Common Name	*Atemoya*	*Campas*	*Chaffey*	*Daniela*	*Fino de Jete*	*Torre1*	*Torre2*	*White*
**1**	Anonaine	n.d.	6.44 (1.17) ^a^	n.d.	n.d.	5.10 (1.06) ^a^	n.d.	5.38 (0.35) ^a^	n.d.
**2**	Asimilobine	n.d.	9.34 (1.1) ^a^	n.d.	8.07 (0.27) ^ab^	6.66 (1.12) ^b^	n.d.	n.d.	2.14 (0.18) ^c^
**3**	Catechin	164.01 (12.21) ^a^	21.28 (2.09) ^bc^	5.41 (0.17) ^d^	6.54 (0.30) ^d^	12.42 (0.82) ^cd^	n.d.	24.5 (2.27) ^bc^	28.79 (1.23) ^bc^
**4**	Liriodenine	3.80 (0.27) ^d^	94.09 (5.89) ^b^	121.21 (5.14) ^a^	n.d.	95.45 (20.44) ^b^	63.55 (7.11) ^c^	n.d.	9.08 (3.45) ^d^
**5**	Quercetin 3-*O*-rutinoside-7-*O*-glucoside	1.73 (0.02) ^bc^	1.06 (0.01) ^cd^	n.d.	n.d.	1.78 (0.20) ^bc^	n.d.	16.16 (1.41) ^a^	2.68 (0.19) ^b^
**6**	Epicatechin	26.31 (1.59) ^b^	12.02 (1.27) ^c^	6.33 (0.17) ^d^	14.29 (0.38) ^c^	10.27 (0.85) ^cd^	n.d.	56.27 (3.24) ^a^	24.24 (1.04) ^b^
**7**	Quercetin 3-*O*-rutinoside-7-*O*-pentoside	105.96 (3.93) ^a^	49.15 (4.70) ^d^	33.97 (2.07) ^e^	66.15 (2.60) ^c^	75.29 (2.14) ^b^	32.25 (2.23) ^ef^	68.24 (3.15) ^bc^	24.61 (0.64) ^f^
**8**	Quercetin 3-*O*-rutinoside	2831.24 (169.56) ^a^	1334.96 (107.40) ^cd^	1648.51 (106.92) ^c^	2107.17 (81.39) ^b^	2234.44 (82.05) ^b^	1015.96 (74.37) ^d^	3167.09 (277.08) ^a^	1127.45 (91.77) ^d^
**10**	Kaempferol-3-Galactoside-7-Rhamnoside	255.12 (19.35) ^b^	76.66 (0.35) ^f^	135.33 (11.78) ^e^	620.98 (1.01) ^a^	165.77 (0.69) ^d^	55.74 (1.21) ^fg^	210.62 (12.09) ^c^	41.84 (4.97) ^g^
**11**	Quercetin-3-*O*-glucoside	529.39 (18.69) ^d^	1016.21 (55.02) ^c^	1281.43 (53.81) ^c^	2593.92 (117.43) ^a^	2388.35 (79.54) ^a^	719.53 (22.55) ^d^	2087.92 (177.80) ^b^	2359.83 (133.23) ^ab^
**12**	Kaempferol-3-*O*-glucoside	6.58 (0.59) ^e^	22.44 (2.28) ^de^	26.73 (1.43) ^de^	337.09 (18.11) ^a^	74.2 (3.78) ^b^	48.16 (3.78) ^c^	25.71 (3.34) ^de^	36.19 (4.24) ^cd^
**13**	Stepharine	157.01 (4.22) ^c^	159.48 (4.93) ^c^	125.92 (11.3) ^d^	105.40 (5.47) ^de^	314.45 (21.63) ^a^	122.62 (7.81) ^d^	202.44 (6.43) ^b^	83.94 (11.38) ^e^
**14**	Apigenin 8-*C*-glucoside	6.02 (0.12) ^d^	9.79 (1.01) ^bc^	6.51 (0.35) ^cd^	10.59 (1.12) ^bc^	8.04 (0.29) ^bcd^	4.94 (0.50) ^d^	17.82 (0.85) ^a^	20.47 (2.92) ^a^
**15**	Lanuginosine	214.52 (10.57) ^de^	793.17 (9.65) ^abc^	1000.27 (71.40) ^a^	154.86 (5.47) ^e^	591.33 (122.16) ^bc^	886.32 (200.42) ^ab^	940.37 (144.81) ^a^	502.21 (108.37) ^cd^
**16**	Luteolin-3-Galactoside-7-Rhamnoside	11.29 (1.20) ^d^	80.48 (0.91) ^b^	122.52 (11.95) ^a^	112.64 (5.60) ^a^	103.26 (9.49) ^a^	120.58 (12.71) ^a^	47.49 (5.86) ^c^	60.98 (5.51) ^bc^
**17**	Luteolin-3-Glucoside-7-Rhamnoside	3.38 (0.34) ^a^	10.38 (1.11) ^b^	14.69 (1.49) ^c^	32.15 (2.22) ^de^	36.69 (3.13) ^de^	52.02 (6.27) ^e^	10.01 (0.48) ^b^	23.82 (2.58) ^d^
**18**	Pronuciferine	121.37 (1.94) ^c^	368.57 (5.42) ^a^	294.84 (8.55) ^b^	88.75 (2.73) ^d^	129.87 (2.86) ^c^	80.42 (12.1) ^d^	42.05 (4.07) ^e^	44.08 (2.80) ^e^

Within the same line, different lowercase letters indicate significant differences at *p* ≤ 0.05 as measured by Tukey’s multiple range test. Letter “a” denotes the highest content. For further statistical analysis information see Supplementary Table S2. For further information about the identified compounds, see Supplementary Table S4 and Supplementary Table S5.

**Table 3:** Radical scavenging activity (ABTS and DPPH) and ferric reducing antioxidant power (FRAP) of the seven extracts of Cherimoya and Atemoya leaves. Values are expressed as mean (±SD) of three experiments carried out in triplicate.

	DPPH	ABTS	FRAP
*Atemoya*	13.51 (0.31) ^cd^	5.01 (0.01) ^e^	14.79 (0.07) ^b^
*Campas*	20.06 (0.47) ^a^	6.92 (0.03) ^d^	13.95 (0.67) ^bc^
*Chaffey*	12.59 (0.75) ^d^	7.17 (0.05) ^c^	12.64 (0.42) ^cd^
*Daniela*	10.31 (0.66) ^e^	4.82 (0.06) ^e^	12.33 (0.88) ^d^
*Fino de Jete*	14.14 (0.58) ^c^	6.87 (0.06) ^d^	14.81 (0.42) ^b^
*Torre1*	7.71 (0.09) ^f^	3.01 (0.05) ^f^	6.98 (0.49) ^e^
*Torre2*	20.21 (0.65) ^a^	9.72 (0.16) ^a^	22.06 (1.39) ^a^
*White*	16.91 (0.13) ^b^	7.99 (0.12) ^b^	14.99 (1.3) ^b^

Data expressed as mmol TE per 100 g of d.wt material. Within the same line, different lowercase letters indicate significant differences at *p* ≤ 0.05 as measured by Tukey’s multiple range test. Letter “a” denotes the highest content. For additional statistical information see Supplementary Table S1.

## Supplementary Materials

The following materials are available online. Table S1: Tukey’s HSD post hoc differences in TPC, tPAC and Antioxidant activity; Table S2: Tukey’s HSD post hoc differences in polyphenolic and alkaloid compounds; Table S3: Tukey’s HSD post hoc differences in antiproliferative activities on HeLa and HepG2; Table S4: Investigated phenolic and alkaloid (gray) compounds identified in the seven extracts of Cherimoya and Atemoya leaves; Table S5: Pearson correlations (ρ) obtained from the comparison of chemical quantifications of alkaloids and antiproliferative activity evaluated on HeLa and HepG2 cell lines. Figure S1: Representative alkaloid (Panel A) and polyphenol (Panel B) profile obtained from the leaves of *A. cherimola*.
