# Supplementary material for: Chemical Profile and Biological Activity of Cherimoya (Annona cherimola Mill.) and Atemoya (Annona atemoya) Leaves
Source: Molecules. 2020 Jun 4;25(11):2612. doi: 10.3390/molecules25112612 (PMC7321297; doi:10.3390/molecules25112612)
Supplement: Supplementary file 1 [file molecules-25-02612-s001.zip › Table S1.docx]

#### Supplementary Table 1: Tukey’s HSD post hoc differences in TPC, tPACs and Antioxidant activity, respectively evaluated by Folin-Ciocalteu, DMAC, ABTS, DPPH and FRAP assay in *Annona cherimola* and *Annona atemoya* leaf extracts. * *p* ≤0.05; ** *p* ≤0.01; *** *p* ≤0.005.

| **Cultivar(s)** | | **FOLIN** | **DMAC** | **ABTS** | **DPPH** | **FRAP** |
| --- | --- | --- | --- | --- | --- | --- |
| *Atemoya* | *Campas* | -52.702 | -78.314*** | -1.917*** | -6.551*** | 0.84 |
|  | *Chaffey* | 16.911 | -5.000** | -2.168*** | 0.927 | 2.152** |
|  | *Daniela* | 76.571 | 33.183*** | 0.175* | 3.213*** | 2.463*** |
|  | *Fino de Jete* | -64.118 | 11.681*** | -1.868*** | -0.622 | -0.019 |
|  | *Torre1* | 223.880*** | 45.493*** | 1.987*** | 5.807*** | 7.809*** |
|  | *Torre2* | -237.531*** | -76.262*** | -4.724*** | -6.696*** | -7.263*** |
|  | *White* | -66.352 | -22.037*** | -2.993*** | -3.397*** | -0.199 |
| *Campas* | *Chaffey* | 69.614 | 73.314*** | -0.251*** | 7.479*** | 1.312 |
|  | *Daniela* | 129.274*** | 111.498*** | 2.092*** | 9.764*** | 1.622* |
|  | *Fino de Jete* | -11.415 | 89.996*** | 0.048 | 5.929*** | -0.859 |
|  | *Torre1* | 276.583*** | 123.808*** | 3.905*** | 12.358*** | 6.968*** |
|  | *Torre2* | -184.829*** | 2.052 | -2.807*** | -0.145 | -8.103*** |
|  | *White* | -13.649 | 56.277*** | -1.076*** | 3.153*** | -1.04 |
| *Chaffey* | *Daniela* | 59.659 | 38.183*** | 2.343*** | 2.285*** | 0.310 |
|  | *Fino de Jete* | -81.030 | 16.681*** | 0.300*** | -1.549*** | -2.172** |
|  | *Torre1* | 206.968*** | 50.494*** | 4.156*** | 4.879*** | 5.656*** |
|  | *Torre2* | -254.443*** | -71.262*** | -2.556*** | -7.624*** | -9.416*** |
|  | *White* | -83.263* | -17.036*** | -0.825*** | -4.325*** | -2.352** |
| *Daniela* | *Fino de Jete* | -140.689*** | -21.501*** | -2.043*** | -3.835*** | -2.482*** |
|  | *Torre1* | 147.309*** | 12.31*** | 1.812*** | 2.594*** | 5.345*** |
|  | *Torre2* | -314.103*** | -109.445*** | -4.899*** | -9.909*** | -9.726*** |
|  | *White* | -142.923*** | -55.22*** | -3.168*** | -6.61*** | -2.662*** |
| *Fino de Jete* | *Torre1* | 287.998*** | 33.812*** | 3.856*** | 6.429*** | 7.828*** |
|  | *Torre2* | -173.413*** | -87.943*** | -2.856*** | -6.074*** | -7.244*** |
|  | *White* | -2.233 | -33.718*** | -1.125*** | -2.775*** | -0.180 |
| *Torre1* | *Torre2* | -461.412*** | -121.756*** | -6.712*** | -12.503*** | -15.072*** |
|  | *White* | -290.232*** | -67.531*** | -4.981*** | -9.204*** | -8.008*** |
| *Torre2* | *White* | 171.179*** | 54.225*** | 1.731*** | 3.298*** | 7.063*** |
