# Supplementary material for: Chemical Profile and Biological Activity of Cherimoya (Annona cherimola Mill.) and Atemoya (Annona atemoya) Leaves
Source: Molecules. 2020 Jun 4;25(11):2612. doi: 10.3390/molecules25112612 (PMC7321297; doi:10.3390/molecules25112612)
Supplement: Supplementary file 1 [file molecules-25-02612-s001.zip › Table S2.docx]

#### Supplementary Table 2: Tukey’s HSD post hoc differences in polyphenolic and alkaloid compounds in the seven extracts of Cherimoya and Atemoya leaves, evaluated by HPLC-DAD-MS/MS. The number in the first line of each column refers to the corresponding compound listed in Table 2. * *p* ≤0.05; ** *p* ≤0.01; *** *p* ≤0.005.
