# Supplementary material for: Chemical Profile and Biological Activity of Cherimoya (Annona cherimola Mill.) and Atemoya (Annona atemoya) Leaves
Source: Molecules. 2020 Jun 4;25(11):2612. doi: 10.3390/molecules25112612 (PMC7321297; doi:10.3390/molecules25112612)
Supplement: Supplementary file 1 [file molecules-25-02612-s001.zip › Table S3.docx]

#### Supplementary Table 3: Tukey’s HSD post hoc differences in antiproliferative activities on HeLa and HepG2 of the seven extracts of Cherimoya and Atemoya leaves, evaluated by MTT assay. * *p* ≤0.05; ** *p* ≤0.01; *** *p* ≤0.005.

| **Varieties** | | **HeLa** | **HepG2** |
| --- | --- | --- | --- |
| *Atemoya* | *Campas* | 1,02*** | 0,63*** |
|  | *Chaffey* | 1,11*** | 1,5*** |
|  | *Daniela* | 0,35*** | -0,85*** |
|  | *Fino de Jete* | -0,09 | -0,06 |
|  | *Torre1* | -2,76*** | -5,33*** |
|  | *Torre2* | -11,87*** | -13,51*** |
|  | *White* | -3,49*** | -2,82*** |
| *Campas* | *Chaffey* | 0,09 | 0,86*** |
|  | *Daniela* | -0,66*** | -1,48*** |
|  | *Fino de Jete* | -1,11*** | -0,7*** |
|  | *Torre1* | -3,78*** | -5,96*** |
|  | *Torre2* | -12,9*** | -14,15*** |
|  | *White* | -4,52*** | -3,45*** |
| *Chaffey* | *Daniela* | -0,75*** | -2,35*** |
|  | *Fino de Jete* | -1,21*** | -1,57*** |
|  | *Torre1* | -3,87*** | -6,83*** |
|  | *Torre2* | -12,99*** | -15,01*** |
|  | *White* | -4,61*** | -4,32*** |
| *Daniela* | *Fino de Jete* | -0,45*** | 0,78*** |
|  | *Torre1* | -3,12*** | -4,47*** |
|  | *Torre2* | -12,23*** | -12,66*** |
|  | *White* | -3,85*** | -1,97*** |
| *Fino de Jete* | *Torre1* | -2,66*** | -5,26*** |
|  | *Torre2* | -11,78*** | -13,44*** |
|  | *White* | -3,4*** | -2,75*** |
| *Torre1* | *Torre2* | -9,11*** | -8,18*** |
|  | *White* | -0,73*** | 2,5*** |
| *Torre2* | *White* | 8,37*** | 10,69*** |
