# Supplementary material for: Chemical Profile and Biological Activity of Cherimoya (Annona cherimola Mill.) and Atemoya (Annona atemoya) Leaves
Source: Molecules. 2020 Jun 4;25(11):2612. doi: 10.3390/molecules25112612 (PMC7321297; doi:10.3390/molecules25112612)
Supplement: Supplementary file 1 [file molecules-25-02612-s001.zip › Table S4.docx]

#### Supplementary Table 4: Investigated phenolic and alkaloid (grey) compounds identified in the seven extracts of Cherimoya and Atemoya leaves: Retention Time (RT), Molecular Weight (MW), fragmentation (m/z) and chemical formula.

| **#** | **Common Name** | **RT** | **MW** | **m/z** | **MS/MS** | **Chemical formula** |
| --- | --- | --- | --- | --- | --- | --- |
| **1** | Anonaine | 12.5 | 265 | 266 | 249 | C_17_H_15_NO_2_ |
| **2** | Asimilobine | 15.6 | 267 | 268 | 251 | C_17_H_17_NO_2_ |
| **3** | Catechin | 20.6 | 290 | 289 | 245 | C_15_H_14_O_6_ |
| **4** | Liriodenine | 21.2 | 275 | 276 | 244 | C_17_H_9_NO_3_ |
| **5** | Quercetin 3-O-rutinoside-7-O-glucoside | 22.3 | 772 | 771 | 609; 463; 301 | C_33_H_40_O_21_ |
| **6** | Epicatechin | 25.5 | 290 | 289 | 245 | C_15_H_14_O_6_ |
| **7** | Quercetin 3-O-rutinoside-7-O-pentoside | 27.2 | 742 | 741 | 609; 300 | C_32_H_38_O_20_ |
| **8** | Quercetin 3-O-rutinoside | 28.1 | 610 | 609 | 301 | C_27_H_30_O_16_ |
| **9** | Unknown | 28.5 | 478 | 477 | 323 |  |
| **10** | Kaempferol-3-Galactoside-7-Rhamnoside | 29.1 | 594 | 593 | 447; 285 | C_27_H_30_O_15_ |
| **11** | Quercetin-3-O-glucoside | 29.1 | 464 | 463 | 301 | C_21_H_20_O_12_ |
| **12** | Kaempferol-3-O-glucoside | 30.1 | 448 | 447 | 284 | C_21_H_20_O_11_ |
| **13** | Stepharine | 31.3 | 297 | 298 | 253; 237 | C_18_H_19_NO_3_ |
| **14** | Apigenin 8-C-glucoside | 32.4 | 448 | 447 | 403; 323 | C_21_H_20_O_10_ |
| **15** | Lanuginosine | 32.7 | 305 | 306 | 274 | C_18_H_11_NO_4_ |
| **16** | Luteolin-3-Galactoside-7-Rhamnoside | 32.9 | 594 | 593 | 447; 285 | C_27_H_30_O_15_ |
| **17** | Luteolin-3-Glucoside-7-Rhamnoside | 33.5 | 594 | 593 | 447; 285 | C_27_H_30_O_15_ |
| **18** | Pronuciferine | 35.7 | 311 | 312 | 295; 263 | C_19_H_21_NO_3_ |
