# Supplementary material for: Chemical Profile and Biological Activity of Cherimoya (Annona cherimola Mill.) and Atemoya (Annona atemoya) Leaves
Source: Molecules. 2020 Jun 4;25(11):2612. doi: 10.3390/molecules25112612 (PMC7321297; doi:10.3390/molecules25112612)
Supplement: Supplementary file 1 [file molecules-25-02612-s001.zip › Table S5.docx]

**Supplementary Table 5**: Pearson correlations (ρ) obtained from the comparison of chemical quantifications of alkaloids and antiproliferative activity evaluated on HeLa and HepG2 cell lines.

|  | **HeLa** | **HepG2** |
| --- | --- | --- |
| Anonaine | -0.391 | -0.36 |
| Asimilobine | -0.991 | -0.919 |
| Liriodenine | -0.603 | -0.422 |
| Stepharine | 0.101 | 0.078 |
| Lanuginosine | 0.347 | 0.357 |
| Pronuciferine | -0.587 | -0.592 |
| tApoC | -0.595 | -0.537 |
| TOxoC | 0.245 | 0.255 |
| tProC | -0.445 | -0.462 |
| TAlkC | 0.053 | 0.056 |
