# Supplementary figures and images for: Chemical Profile and Biological Activity of Cherimoya (Annona cherimola Mill.) and Atemoya (Annona atemoya) Leaves
Source: Molecules. 2020 Jun 4;25(11):2612. doi: 10.3390/molecules25112612 (PMC7321297; doi:10.3390/molecules25112612)

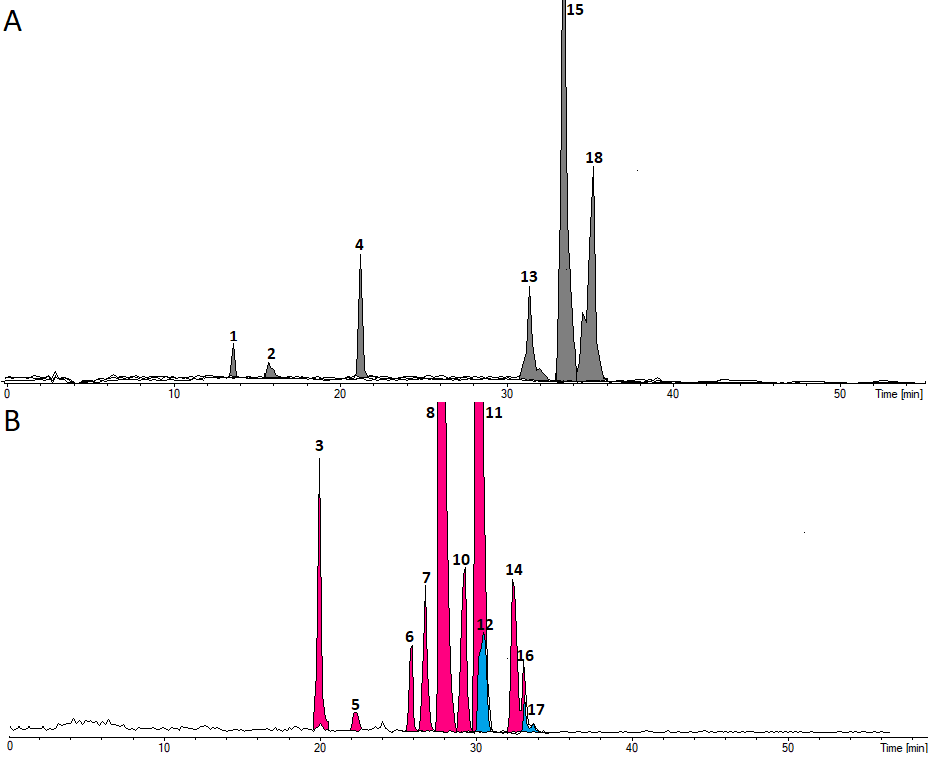

Supplement: Supplementary file 1 [file molecules-25-02612-s001.zip › Figure S1.tif]
